# Open access, moving to the fore

**DOI:** 10.1186/1742-4690-9-66

**Published:** 2012-08-13

**Authors:** Kuan-Teh Jeang

**Affiliations:** 1 The National Institutes of Health, Bethesda, MD, USA

## Abstract

Nine years after its founding, *Retrovirology* has moved to the forefront of virology journals in Impact Factor.

## 

In 2004, during the early days of Open Access, I had the opportunity to start *Retrovirology* employing the then “new way” of publishing [1]. *Retrovirology* was not the first journal that I helped found. Ten years earlier, in 1994, I was one of nine editors, led by Dr. Chuan C. Chang, who started the *Journal of Biomedical Science*[2]. The *Journal of Biomedical Science* originated as a subscription-based journal; thus, when *Retrovirology* began I understood the difference between a publishing model based on subscription (readers/subscribing libraries and institutions pay) versus Open Access (authors pay, and all articles are freely accessible by readers).

At the outset, there were two challenges to *Retrovirology’s* success. The first was whether Open Access would be a sustainable business model. In those days, this was an unknown. Today, the increasing popularity of journals like *PLoS ONENature’s Scientific Reports (http://www.nature.com/srep/index.html)Cell Reports (http://www.cellreports.cell.com)Cell and Bioscience*[3], *Journal of the International AIDS Society* (*http://www.jiasociety.org/index.php/jias*), and the recent migration of journals such as *EMBO Molecular Medicine* from a subscription to an Open Access format indicate that the latter business model has achieved financial traction, if not overt profitability.

The second challenge was an early notion held by some that Open Access journals would publish lower “quality” science with inherently less “visible” findings. A few contentious colleagues even insisted, “*Retrovirology* will never reach the Impact Factors of the *Journal of Virology*, *Virology*, and the *Journal of General Virology*!” In retrospect, they were wrong; *Retrovirology* achieved and surpassed those metrics. Indeed, in the 2011 tabulation of Impact Factor and Immediacy Index, *Retrovirology* placed ahead of the *Journal of Infectious Diseases*, *AIDS*, *JAIDS*, *J Virol*., *Virology*, *J Gen Virology*; and two established standards of molecular biology and biochemistry, the *Journal of Molecular Biology* and the *Journal of Biological Chemistry* (Figure 1). Of interest, amongst these journals, *Retrovirology* is the only Open Access journal. This means that only in *Retrovirology* are your papers immediately available for all to read, the very day that they are published, in full text form without the readers being encumbered by subscription fees. This Open Access feature may explain the large advantage in Immediacy Index for papers published in *Retrovirology* over the next-ranked journal, *the Journal of Infectious Diseases* (Figure 1).

**Figure 1:**
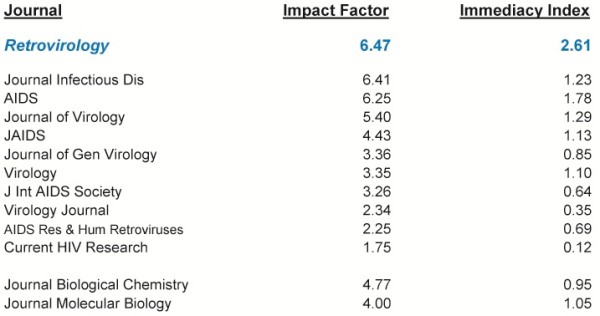
**Impact factor and immediacy index of*Retrovirology*and the indicated journals.** The data are from the 2011 Journal Citation Reports (ISI Web of Knowledge, Thomson-Reuters).

Impact Factor and Immediacy Index are two of several proxies of a journal’s quality, and one should interpret cautiously their meaning [4]. Arguably, a better measure is to ask how a journal’s papers have made a difference in its field. In this respect, a significant example can be drawn from six *Retrovirology* papers published in December 2010 that were the first to pivotally correct the then held belief that XMRV was an etiological cause of Chronic Fatigue Syndrome (CFS) [5-10]. In that instance, *Retrovirology’s* Open Access format was particularly instrumental in permitting interested individuals, who were not career scientists, to freely, rapidly, and fully access those paradigm-changing peer-reviewed publications.

Increasing data support the absence of inherent reasons for qualitative difference between papers published in subscription versus Open Access journals [11]. In my view, whether a journal moves to the fore is dictated by the diligence and dedication of its editorial board. *Retrovirology’s* strong progress forward is owed to the efforts of its board members (http://www.retrovirology.com/about/edboard).
